# Phosphorylation of interleukin (IL)-24 is required for mediating its anti-cancer activity

**DOI:** 10.18632/oncotarget.3977

**Published:** 2015-05-18

**Authors:** Janani Panneerselvam, Manish Shanker, Jiankang Jin, Cynthia D. Branch, Ranganayaki Muralidharan, Yan D. Zhao, Sunil Chada, Anupama Munshi, Rajagopal Ramesh

**Affiliations:** ^1^ Department of Pathology, The University of Oklahoma Health Sciences Center, Oklahoma City, Oklahoma, USA; ^2^ Department of Radiation Oncology, The University of Oklahoma Health Sciences Center, Oklahoma City, Oklahoma, USA; ^3^ Department of Biostatistics and Epidemiology, The University of Oklahoma Health Sciences Center, Oklahoma City, Oklahoma, USA; ^4^ Stephenson Cancer Center, The University of Oklahoma Health Sciences Center, Oklahoma City, Oklahoma, USA; ^5^ Graduate Program in Biomedical Sciences, The University of Oklahoma Health Sciences Center, Oklahoma City, Oklahoma, USA; ^6^ Department of Thoracic & Cardiovascular Surgery, The University of Texas M.D. Anderson Cancer Center, Houston, Texas, USA; ^7^ DNASolve, Houston, Texas, USA; ^8^ The University of Texas Dental School, Houston, Texas, USA; ^9^ Department of Gastrointestinal Oncology, The University of Texas MD Anderson Cancer Center, Houston, Texas, USA; ^10^ Department of Plastic Surgery, The University of Texas MD Anderson Cancer Center, Houston, Texas, USA

**Keywords:** IL-24, phosphorylation, lung cancer, cytokine

## Abstract

Interleukin (*IL*)-*24* is a tumor suppressor/cytokine gene that undergoes post-translational modifications (PTMs). Glycosylation and ubiquitination are important for IL-24 protein stabilization and degradation respectively. Little is known about IL-24 protein phosphorylation and its role in IL-24-mediated anti-tumor activities. In this study we conducted molecular studies to determine whether IL-24 phosphorylation is important for IL-24-mediated anti-cancer activity.

Human H1299 lung tumor cell line that was stably transfected with a doxycycline (DOX)-inducible (Tet-on) plasmid vector carrying the cDNA of *IL-24*-wild-type (IL-24^wt^) or *IL-24* with all five phosphorylation sites replaced (IL-24^mt^) was used in the present study. Inhibition of tumor cell proliferation, cell migration and invasion, and induction of G2/M cell cycle arrest was observed in DOX-induced IL-24^wt^-expressing cells but not in IL-24^mt^-expressing cells. Secretion of IL-24^mt^ protein was greatly reduced compared to IL-24^wt^ protein. Further, IL-24^wt^ and IL-24^mt^ proteins markedly differed in their subcellular organelle localization. IL-24^wt^ but not IL-24^mt^ inhibited the AKT/mTOR signaling pathway. SiRNA-mediated AKT knockdown and overexpression of myristolyated AKT protein confirmed that IL-24^wt^ but not IL-24^mt^ mediated its anti-cancer activity by inhibiting the AKT signaling pathway.

Our results demonstrate that IL-24 phosphorylation is required for inhibiting the AKT/mTOR signaling pathway and exerting its anti-cancer activities.

## INTRODUCTION

Interleukin (*IL)-24* is a novel tumor suppressor and a member of the IL-10 cytokine superfamily [1, 2]. Endogenous IL-24 protein expression is detectable in the peripheral blood mononuclear cells (PBMCs), T- and B-cells and in melanocytes [2, 3]. However, IL-24 protein expression is lost in a majority of cancer cells of human origin [1, 2–4]. Previous studies from our laboratory and others have demonstrated that IL-24 has anti-tumor, anti-metastatic, and anti-angiogenic activities [3–8]. Further, studies have also shown that IL-24 is a pro-inflammatory cytokine and stimulates the Th1-type immune response [2, 9], and is subject to post-translational modifications (PTMs), including phosphorylation, glycosylation, and ubiquitination [9–11]. IL-24 is reported to interact with protein kinase [12]. However, whether phosphorylation is required for IL-24-mediated antitumor activities is unknown.

In the present study, we investigated whether IL-24 phosphorylation is required for antitumor activities. The human *IL-24* DNA sequence has five potential phosphorylation sites: Serine (Ser) 88, 101, and 161, and Threonine (Thr) 111 and 133. Using molecular techniques, we replaced all of the five phosphorylation sites, producing a mutant (IL-24^mt^). We compared IL-24^mt^ with wild-type IL-24 (IL-24^wt^). New to science, our data show that IL-24 phosphorylation is required for IL-24-mediated anti-cancer activities.

The present study provides a platform for identifying the phosphorylation site(s) critical for IL-24 to function as an anti-cancer drug. Studies investigating the molecular mechanisms of IL-24 phosphorylation are also warranted.

## RESULTS

### IL-24^wt^ and IL-24^mt^ have different protein banding patterns and cellular localization

**Figure 1:**
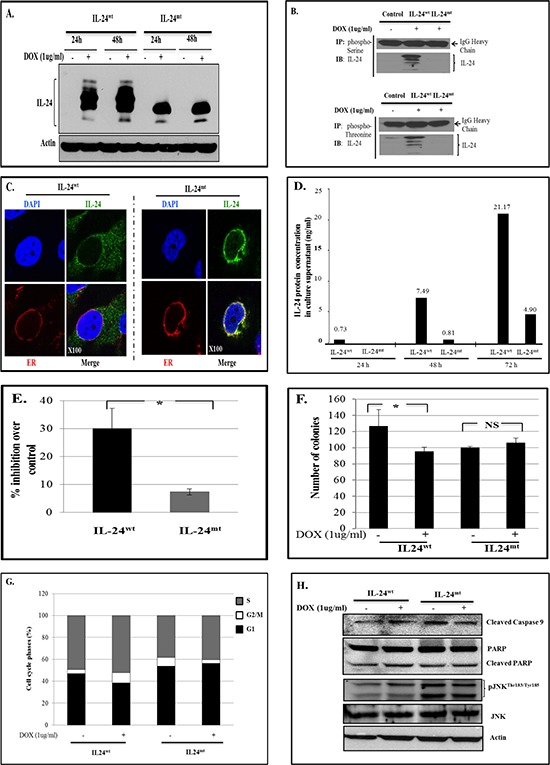
IL-24^wt^ and IL-24^mt^ have different protein banding patterns **A.** Western blotting showed that IL-24^wt^ and IL-24^mt^ protein banding patterns differed following DOX treatment of H1299-*IL-24^wt^* and H1299-*IL-24^mt^* cells. Cells that did not receive DOX treatment served as controls. **B.** Cell lysates from DOX-treated H1299-*IL24^wt^* and H1299-*IL24^mt^* were immunoprecipitated (IP) with phosphorylated Serine or Threonine antibody and immunoblotted (IB) with human IL-24 antibody. IL-24 protein was detected in H1299-*IL-24^mt^* cell lysate, but not in H1299-IL-24*^mt^* cell lysate. This shows that only wild-type IL-24 protein is phosphorylated. IgG protein band served as internal protein loading control. **C.** Immunofluorescence studies showed that IL-24^wt^ protein was uniformly distributed in the cytoplasm, with some localized in the endoplasmic reticulum (ER) of the cell. In contrast, IL-24^mt^ protein was mostly localized in the ER, with little distributed in the cytoplasm of the cell. *Magnification*, x100. **D.** IL-24 protein level was markedly low in the culture supernatant collected from DOX-treated H1299-*IL24^mt^* cells compared with the IL-24 protein level in the supernatant from DOX-treated H1299-*IL24^wt^* cells, as determined by ELISA. Cell culture supernatant from untreated cells served as a negative control. The number above the bar indicates the protein concentration (ng/ml). **E.** Expression of IL-24^wt^ following DOX treatment greatly reduced cell viability of H1299 cells, compared with cells expressing IL-24^mt^ at 72 h. **F.** A colony formation assay on soft agar demonstrated that H1299-*IL-24^wt^* cells formed fewer colonies than H1299-*IL-24^mt^* when treated with DOX. **G.** Cell cycle analysis showed that only IL-24^wt^ induced G2/M cell-cycle arrest at 48 h after DOX treatment. **H.** IL-24^wt^ activated caspase-9, PARP and pJNK^Thr183/Tyr185^ in H1299 cells at 48 h after DOX treatment, while IL-24^mt^ did not. Beta actin was detected as protein loading control. *denotes *P* < 0.05. “*NS*” denotes *Not Significant*.

IL-24^wt^-expressing H1299 cells showed a typical expression pattern [3, 11] with multiple 17 Kd to 26 Kd bands, representing different post-translational modification and maturation stages of IL-24 protein (Figure 1A). However, IL-24^mt^-expressing cells showed a single 19–20 Kd protein band, suggesting that phosphorylation regulates IL-24 protein maturation.

We used immunoprecipitation to determine the phosphorylation status of IL-24^wt^ and IL-24^mt^ protein. We detected 26 Kd bands in sample lysates prepared from IL-24^wt^-expressing cells, reflecting IL-24^wt^ protein (Figure 1B). No 26 Kd band was observed in lysates prepared from IL-24^mt^-expressing cells, indicating that IL-24^mt^ protein is not phosphorylated.

Since exogenous IL-24^wt^ protein expression is detectable in the cytoplasm, endoplasmic reticulum (ER), and secretory organelles [3, 13, 14], we investigated whether phosphorylation affected IL-24 cellular localization. Confocal microscopy and immunofluorescence revealed that IL-24^wt^ protein localized to the cytoplasm, ER, and Golgi. However, IL-24^mt^ protein was primarily localized to the perinuclear area and overlapped with ER (Figure 1C). These results suggest that phosphorylation of IL-24 protein is important for proper subcellular localization and trafficking in the cells.

To determine whether these results were unique to the stably-transfected H1299 single cell clone, we transiently transfected naïve H1299 cells with pcDNA3.1 plasmid DNA vector carrying *IL-24^wt^* and *IL-24^mt^* cDNA under the control of constitutively active cytomegalovirus (CMV) promoter (Supplementary Figure 1A). Western blotting showed that IL-24^wt^- and IL-24^mt^-expressing cells had different banding, irrespective of the time of analysis (Supplementary Figure 1B). We further tested IL-24 protein expression in a human melanoma (MeWo) cell line and compared to protein expression in H1299 cells by transient transfection using the pcDNA3.1-*IL24^wt^* or -*IL24^mt^* plasmids. MeWo and H1299 cells showed the same differences in the protein banding for IL-24^wt^ and IL-24^mt^ (Supplementary Figure 1C). Protein localization study results concurred with the results that were observed with the stable H1299 cell line (Supplementary Figure 1D). Thus, the banding pattern and organelle localization is not cell-line-specific, and protein phosphorylation is important for IL-24 protein maturation, localization, and secretion.

### IL-24^mt^ protein secretion is reduced in doxycycline-induced lung cancer cells

IL-24 protein is known to be secreted [6, 13, 14]. Phosphorylation is reported to regulate protein secretion and function [15, 16]. To determine whether phosphorylation modulates IL-24 protein secretion, we used ELISA to analyze IL-24 protein in cell culture supernatants from DOX-treated H1299-*IL-24^wt^* and H1299-*IL-24^mt^* cells. We detected IL-24 protein in supernatants collected from both cell types (Figure 1D). However, there was markedly less IL-24 protein in IL-24^mt^-expressing cells than in IL-24^wt-^expressing cells (Figure 1D). These results demonstrate that phosphorylation regulates IL-24 protein secretion. Modification of the phosphorylation sites resulted in intracellular retention of IL-24 protein, leading to reduced secretion. Our western blot data (Supplementary Figure 1C) also showed reduced IL-24 protein in the culture supernatant from H1299 and MeWo cells.

### IL-24^wt^ expression reduces cell viability and colony formation

Exogenous IL-24 expression was shown to inhibit tumor cell viability [3, 6, 9]. Therefore, we performed cell viability assays to determine whether modification of the phosphorylation sites in IL-24^wt^ influences its ability to inhibit growth. IL-24^wt^-expressing H1299 cells were significantly less viable than IL-24^mt^-expressing cells (*P* < 0.05; Figure 1E). We also conducted colony formation assays on soft agar. IL-24^wt^ expression inhibited cell growth, with fewer IL-24^wt^-expressing cell colonies than controls (25% reduction; Figure 1F). In contrast, IL-24^mt^-expressing H1299 cells had slightly more colonies than controls (Figure 1F).

### IL-24^wt^ induces cell cycle arrest

Previous studies reported that IL-24^wt^ expression in cancer cells results in cell cycle arrest, leading to apoptosis [3, 5, 17]. We therefore investigated whether IL-24^wt^ and IL-24^mt^ expression perturbed cell cycle progression in H1299 cells. IL-24^wt^ expression increased the number of cells in the G2/M phase (9.3%) compared with controls (3.65%; Figure 1G). IL-24^mt^ expression decreased the number of cells in the G2/M phase (3.4%) compared with controls (8.05%). These results demonstrate that phosphorylation is required for IL-24^wt^-mediated G2/M-phase cell-cycle inhibition.

### IL-24^wt^ activates caspase-9, PARP, and JNK

Our studies and others have demonstrated that adenovirus (Ad)-IL-24 induces apoptosis in cancer cells by activating caspase-9, poly (ADP ribose) polymerase (PARP), and JNK [3, 17–19]. In the present study, we determined the status of these proteins in IL-24^wt^- and IL-24^mt^-expressing H1299 cells. We observed activated caspase-9, PARP, and phosphorylated (p) JNK^Thr183/Tyr185^ only in IL-24^wt^-expressing cells, indicating that phosphorylation is required for the pro-apoptotic activity of IL-24 (Figure 1H).

### IL-24^wt^ inhibits tumor cell migration and cell invasion

We previously demonstrated that lung cancer cells expressing exogenous wild-type IL-24 migrate and invade less [20]. Here, we performed a scratch assay to evaluate whether cell migration and invasion were altered by modification of the phosphorylation sites in IL-24^wt^ cells.

**Figure 2:**
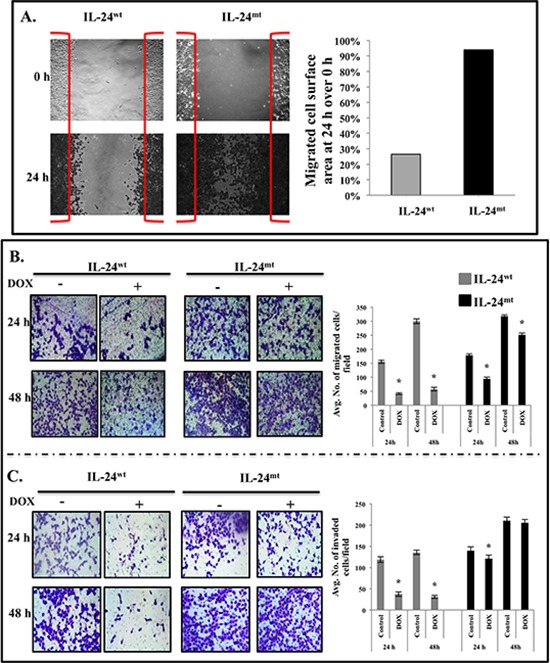
IL-24^wt^, but not IL-24^mt^, inhibits cell migration and invasion **A.** Scratch assay showed that IL-24^wt^-expressing H1299 cells migrated less than IL-24^mt^-expressing H1299 cells. **B.** IL-24^wt^ produced a greater inhibitory effect on cell migration than did IL-24^mt^ at 24 h and 48 h after DOX treatment. **C.** Matrigel invasion assay showed that IL-24^wt^ inhibited cell invasion to a greater extent than IL-24^mt^ at 24 h and 48 h after DOX treatment. Cells that did not receive DOX treatment served as controls. Results shown are the means ± *SD* of three independent experiments. *P < 0.05.

IL-24^wt^-expressing cells migrated into 26.5% of the scratched area, whereas IL-24^mt^-expressing cells migrated into 94% of the scratched area (Figure 2A). To further confirm that lack of IL-24 phosphorylation abrogates the inhibition of cell migration, cell migration assays were performed. IL-24^wt^ expression reduced tumor cell migration, compared with controls, at 24 h and 48 h (*P* = 0.01; Figure 2B). IL-24^mt^-expressing cells also migrated less than controls, although to a lesser degree than IL-24^wt^-expressing cells (Figure 2B).

We next performed a Matrigel assay, which mimics the invasive process of tumor cells travelling through basement membrane components. A significant reduction in cell invasion was observed only in IL-24^wt^-expressing cells (*P* = 0.001; Figure 2C). These results demonstrate that phosphorylation is required for IL-24-mediated inhibition of tumor cell migration and invasion.

### IL-24^wt^ inhibits the AKT-mTOR signaling pathway

**Figure 3:**
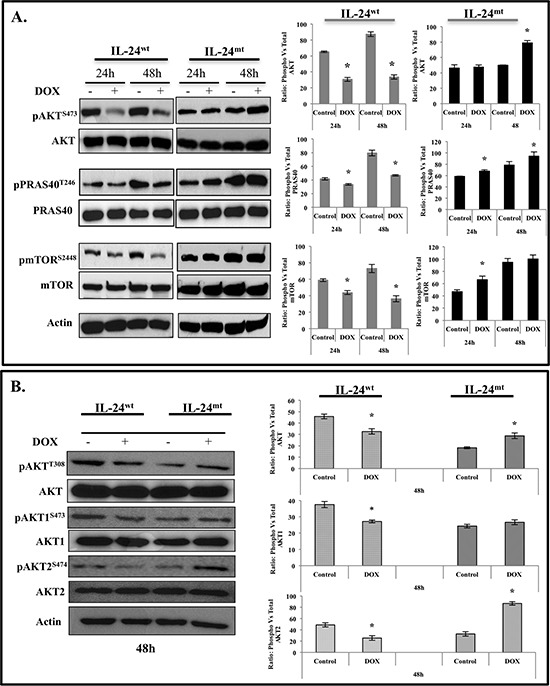
IL-24^wt^, but not IL-24^mt^, inhibits the AKT-mTOR signaling pathway Cell lysates prepared from DOX-treated H1299-*IL-24^wt^* and H1299-*IL-24^mt^* cells were analyzed for proteins associated with the AKT signaling pathway and changes in protein expression quantified by Western blotting. **A.** IL-24^wt^, but not IL-24^mt^, significantly reduced the expression of phosphorylated (p) AKT^S473^, pPRAS40^T246^, and pmTOR^S2448^ proteins at the two time points tested. **B.** Analysis for changes in the expression of AKT isoforms demonstrated that IL-24^wt^ significantly reduced the expression of pAKT^T308^, pAKT1^S473^, and pAKT2^S474^. Increased expression of all of the AKT isoforms was observed in IL-24^mt^-expressing H1299 cells. Beta actin was detected as protein loading control. *denotes *P* < 0.05.

We investigated the signaling mechanism by which IL-24 phosphorylation regulates its anti-tumor activity. Reverse phase protein array (RPPA) analysis [21, 22] on IL-24^wt^ and IL-24^mt^ cell lysates indicated different expression levels of proteins involved in the AKT-mammalian target of rapamycin (mTOR) pathway (data not shown). Thus, we investigated the regulation of the AKT-mTOR pathway by IL-24. Induction of IL-24^wt^ in H1299-*IL24^wt^* cells significantly decreased pAKT^S473^ protein expression at 24 h and 48 h (Figure 3A). Expression of a critical downstream target of AKT, pPRAS40^T246^, was also reduced upon IL-24^wt^ induction (Figure 3A). Furthermore, IL-24^wt^ markedly reduced pmTOR^S2448^ expression at 24 and 48 h (Figure 3A). The expression of IL-24^mt^ protein in H1299-*IL24* cells produced no change in pAKT^S473^ expression at 24 h (Figure 3A). However, we observed significantly increased expression at 48 h (*P* < 0.05; Figure 3A). pPRAS40^T246^ expression increased at 24 h and 48 h, while pmTOR^S2448^ expression only increased at 24 h.

We further evaluated the effect of IL-24 phosphorylation on pAKT^T308^ expression and on AKT isoforms AKT1 and 2. AKT1 and 2 are ubiquitously expressed and individually contribute to cell survival, proliferation, and metastasis via different mechanisms [23]. In IL-24^wt^-expressing cells, we observed a marked reduction in pAKT^T308^, pAKT1^S473^, and pAKT2^S474^ expression at 48 h, compared with controls. IL-24^mt^ expression increased pAKT^T308^ and pAKT2^S474^, with no change in pAKT1^S473^, suggesting that IL-24 phosphorylation is involved in the regulation of AKT isomers (Figure 3B).

### IL-24 mediates anti-tumor activity by suppressing AKT and AKT-mediated PRAS40 phosphorylation

**Figure 4:**
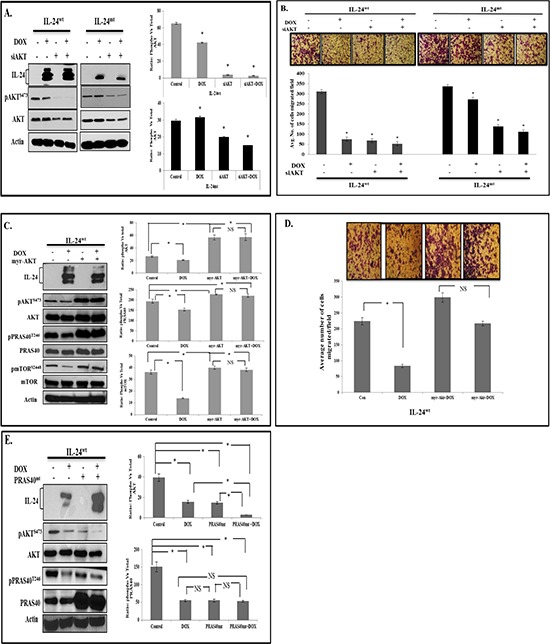
IL-24 mediates anti-tumor activity by suppressing AKT and AKT-mediated PRAS40 phosphorylation H1299-*IL-24^wt^* and H1299-*IL-24^mt^* cells were transfected with AKT siRNA, or not transfected, followed by treatment with or without DOX (1 μg/ml). Then, cells were subjected to molecular analysis at 48 h after DOX treatment. **A.** Western blotting showed that IL-24^wt^ plus AKT siRNA produced a greater inhibitory effect on pAKT^S473^ expression than IL-24^mt^ plus siAKT. **B.** Cell migration assay results correlated with Western blotting and showed that IL-24^wt^ plus siAKT produced inhibited cell migration more than IL-24^mt^ plus siAKT. **C.** H1299- *IL-24^wt^* cells were either not transfected, or transfected with myr-AKT plasmid, followed by treatment with or without DOX (1 μg/ml) and subjected to molecular analysis at 48 h after treatment. Expression of myr-AKT abrogated the inhibitory effect of IL-24^wt^ on AKT and its downstream targets, PRAS40 and mTOR. **D.** IL-24^wt^-mediated inhibition of cell migration was reduced when myr-AKT was expressed. **E.** Phosphorylation of PRAS40^T246^ and AKT^S473^ was significantly reduced in IL-24^wt^-expressing and PRAS40^mt^-expressing cells compared with controls. However, greater inhibition of pPRAS40^T246^ and pAKT^S473^ was observed when IL-24^wt^ and PRAS40^mt^ were co-expressed. Control cells did not receive any treatment. Beta actin was detected as protein loading control. *denotes *P* < 0.05. “*NS*” denotes *Not Significant*.

To evaluate whether IL-24 mediates its anti-tumor function by inhibiting AKT activation and phosphorylation is required for AKT inhibition, we knocked down AKT in H1299-*IL-24* cells with si-AKT. pAKT^S473^ expression and cell migration were significantly lower in IL-24^wt^-expressing cells than controls (Figure 4A, 4B). Si-AKT knockdown mimicked the effect of IL-24^wt^ expression on cell migration. We observed enhanced cell migration inhibition upon combining IL-24^wt^ and si-AKT, compared with the individual treatments (Figure 4A, 4B). IL-24^mt^-expressing cells showed increased pAKT^S473^ expression that was, however, reduced in the presence of si-AKT to levels similar to those observed in H1299-*IL-24^wt^* cells (Figure 4A, 4B). IL-24^mt^, unlike IL-24^wt^, showed reduced cell migration inhibition compared with controls. The combination of treatment inhibited cell migration more than the individual treatments did. Together, our data show that IL-24^wt^ mediates its anti-tumor activity by regulating AKT, and, importantly, that IL-24^wt^ must be phosphorylated to regulate AKT.

We also examined whether IL-24^wt^ could mediate its tumor suppressor effects when AKT was overexpressed. Constitutive AKT expression was achieved by transfecting H1299-*IL24^wt^* cells with myr-AKT plasmid. We observed significantly increased pAKT^S473^, pPRAS40^T246^, and mTOR^S2448^ expression in myr-AKT-expressing cells compared with vector controls (Figure 4C). However, we observed no noticeable IL-24^wt-^mediated inhibition of AKT or its downstream signaling proteins in myr- AKT-expressing cells. Furthermore, expression of myr-AKT abrogated the IL-24^wt^-mediated inhibition of cell migration compared with IL-24^wt^-mediated inhibition in the absence of myr-AKT (*P* < 0.05; Figure 4D). Our results confirm that the regulation of AKT signaling and phosphorylation are crucial for IL-24^wt^ to produce anti-tumor effects.

AKT-mediated phosphorylation of PRAS40 at Threonine (T) 246 inactivates PRAS40 and influences mTOR activation [24]. Our myr-AKT study results concurred with this report. We used PRAS40^mt^ plasmid to further test whether reduced PRAS40 expression in IL-24^wt^-expressing cells occurs via AKT. The plasmid expresses PRAS40 protein, in which the T is replaced with Alanine (A) at amino acid 246 (^T^246^A^), such that AKT cannot phosphorylate PRAS40, resulting in reduced pPRAS40 expression and mTOR activation. We anticipated that induction of IL-24^wt^, along with mutated PRAS40 expression, would show greater reduction in pPRAS40 expression. PPRAS40^T246^ expression was significantly reduced in mutant PRAS40^T246A^ plasmid-transfected cells compared with controls (*P* < 0.05; Figure 4E). However, pPRAS40 expression was further reduced when combined with IL-24^wt^, comparable to our findings from cells expressing IL-24^wt^ alone (Figure 4E). Our results show that IL-24^wt^ inhibits AKT-mediated phosphorylation and PRAS40 inactivation to execute its anti-tumor activity.

### IL-24^wt^ modulates PRAS40-Raptor interaction

**Figure 5:**
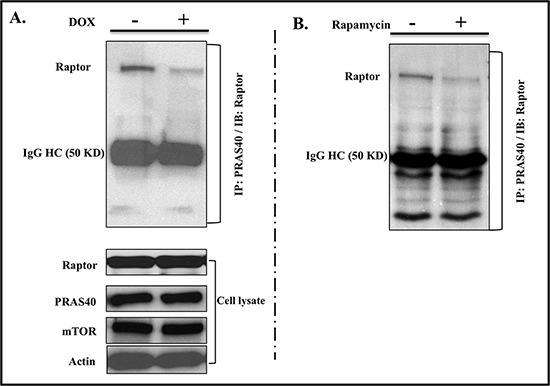
IL-24^wt^ modulates PRAS40 and Raptor interaction Immunoprecipitation studies showed that Raptor protein bound to PRAS40 was markedly reduced in **A.** IL-24^wt^-expressing cells and **B.** rapamycin-treated cells, compared with untreated controls. Total proteins for Raptor, PRAS40, and mTOR were detected in cell lysates to ensure that the reduced Raptor binding was not due to a reduction in total protein levels. Beta actin was detected as protein loading control.

AKT is known to inactivate PRAS40 via phosphorylation, thereby disrupting PRAS40-mTORC1 binding and removing PRAS40 inhibition of mTORC1 [24, 25]. PRAS40 binds to mTORC1 primarily through interactions with Raptor. Our results showed that IL-24^wt^ expression reduced AKT-mediated PRAS40 inactivation. IL-24^wt^ expression might, then, increase PRAS40 inhibition of mTORC1 by increasing the tight interaction between PRAS40 and Raptor. To test this, we treated H1299-*IL24^wt^* cells with DOX for 48 h. The cytosolic extracts were immunoprecipitated with PRAS40 and immunoblotted for Raptor. We observed a marked reduction in Raptor binding to PRAS40 upon IL-24^wt^ induction (Figure 5A). Similar results were observed in H1299-*IL24^wt^* cells that were treated with mTORC1 inhibitor rapamycin (100 nM) for 48 h (Figure 5B), suggesting that IL-24^wt^ functions similarly to rapamycin in inhibiting mTORC1.

### IL-24^wt^ alters the expression of downstream targets of AKT

**Figure 6:**
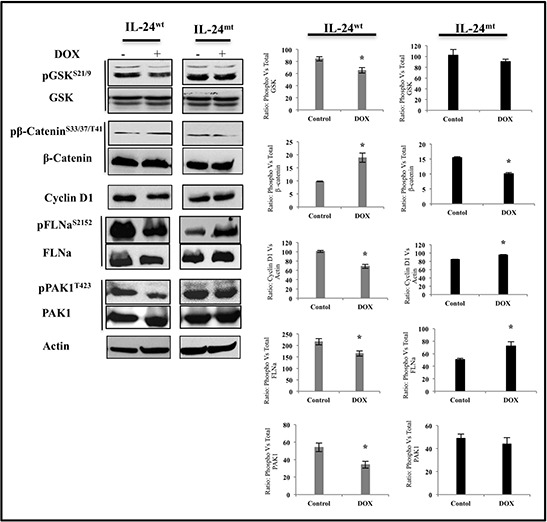
IL-24^wt^ alters the expression of downstream targets of AKT H1299-*IL-24^wt^* and H1299-*IL-24^mt^* cells treated with DOX (1 μg/ml) were subjected to molecular analysis at 48 h after treatment. Control cells did not receive any treatment. Only IL-24^wt^ reduced the protein expression of pGSK^S21/9^, pFLNa^S2152^, pPAK1^T423^, and cyclin D1. Expression of pβ-catenin^S33/37/T41^, however, was increased in IL-24^wt^-, but not in IL-24^mt^-, expressing cells. Beta actin was detected as protein loading control. *denotes *P*-value < 0.05.

We next determined the expression of proteins that are downstream of AKT in H1299 cells after induction of IL-24^wt/mt^ protein expression. IL-24^wt^ significantly reduced pGSK-3^S21/9^ and cyclin D1 and markedly increased pβ-catenin^S33/37/T41^ expression, compared with controls (Figure 6). Filamin A (FLNa) and p21-activated kinase 1 (PAK1) are also downstream targets of AKT. Expression of pFLNa^S2152^ and its downstream target pPAK1^T423^ were significantly reduced in IL-24^wt^-expressing cells (Figure 6). However, IL-24^mt^-expressing cells exhibited significant reduction of pβ- catenin^S33/37/T41^, and increased cyclin D1, pFLNa^S2152^, and pPAK1^T423^, with no change in pGSK-3^S21/9^ protein expression, compared with controls (Figure 6). Our results demonstrate that only IL-24^wt^ effectively inhibits AKT and its downstream target proteins.

## DISCUSSION

Protein phosphorylation is a common PTM in mammalian cells, playing a crucial role in regulating protein functions [16, 26]. Upon phosphorylation, proteins regulate critical cellular processes, such as the cell cycle, apoptosis, signal transduction, metabolism, survival, proliferation, and development [16, 26, 27]. In this study, we investigated whether phosphorylation of IL-24 is critical for anti-tumor activity. For this, we generated DOX-inducible H1299-*IL24^wt^* and H1299-*IL24^mt^* cells. We chose H1299 cells because these cells do not express endogenous IL-24 protein and lack IL-24 receptors [6, 13]. Thus, any anti-tumor activity observed against H1299 cells would be solely attributable to the exogenous IL-24 that is expressed intracellularly when induced by DOX and not confounded by secreted IL-24 [6, 13].

We demonstrated for the first time that lack of IL-24 phosphorylation affects its subcellular localization, secretion, and anti-tumor activity (Figures 1 and 2). Further, transient transfection studies revealed that our results were not cell-type-specific or attributable to the use of stable clones (Supplementary Figure 1). Other tumor suppressor proteins, including p53, LKB1, and von Hippel-Lindau proteins, require phosphorylation to function [28–31]. Similarly, phosphorylation of the herpes simplex virus type 1 ICP0 protein is necessary for its subcellular localization and efficient function [32]. Phosphorylation is also known to regulate protein secretion. Oh *et al*. [33] demonstrated that HMGB1phosphorylation is critical for HMGB1 protein secretion.

Functional studies showed that only IL-24^wt^ significantly inhibited cell growth and colony formation, induced G2/M cell-cycle arrest, and activated caspase-9, PARP, and JNK, indicating that phosphorylation is required for IL-24 to exert anti-tumor effects (Figure 1). Zhao *et al*. [34] showed that AMPK protein phosphorylation was essential for inducing G1 cell cycle arrest and inhibition of cell proliferation and colony formation in nasopharyngeal carcinoma cells. Similarly, stress-activated protein kinases (SAPK)-mediated phosphorylation of p53 induces nuclear p53 accumulation and transcriptional activation of proteins involved in cell cycle arrest and apoptosis [35]. These findings are consistent with our results showing that subcellular localization, protein secretion, and the tumor-suppressive function of IL-24 is modulated by phosphorylation.

Tumor cell migration and invasion play important roles in lung cancer progression and metastasis [36]. We and others have shown that IL-24 inhibits metastasis by inhibiting cell migration and invasion [20]. Therefore, we focused on investigating the importance of IL-24 phosphorylation in the inhibition of migration and invasion of H1299-*IL24* cells *in vitro*. Migration and invasion were significantly reduced only in IL-24^wt^-expressing cells (Figure 2). Consistent with our data, phosphorylation of the human estrogen receptor-beta at Serine 105 resulted in inhibition of breast cancer cell migration and invasion [37]. Our data reveal that IL-24 phosphorylation is important for its anti-metastatic function.

We next determined the molecular signaling mechanism by which IL-24^mt^ and IL-24^wt^ differed. The AKT/mTOR signaling pathway is associated with the development of human cancers, including lung cancer [38]. AKT is an essential downstream component of PI3K-mediated oncogenic signaling and provides critical cell survival signals for tumor progression by phosphorylating several proteins involved in cell cycle regulation and pro-apoptotic factors [38]. Studies showed that pharmacologic and biological inhibition of AKT/mTOR signaling suppressed cancer cell migration, invasion, and metastasis [38]. In parallel, we previously reported that IL-24 can negatively regulate the PI3K/AKT signaling pathway in Ad-IL24-transduced lung and breast cancer cells [39]. Consistent with these reports, only IL-24^wt^ significantly inhibited the expression of AKT isoforms and their downstream signaling proteins (Figure 3). Studies using si-AKT and myr-AKT plasmid DNA demonstrated that IL-24^wt^ specifically inhibits AKT (Figure 4A, 4B). This inhibition activated PRAS40, as evidenced by its reduced phosphorylation at Threonine 246 residue, and mTOR inhibition. AKT-mediated PRAS40 phosphorylation at Threonine 246 promotes PRAS40 binding to 14-3-3 protein complex, thereby weakening PRAS40 interaction with mTORC1, resulting in mTOR activation [25]. Further evidence for the involvement of PRAS40 in AKT/mTOR pathway inhibition was demonstrated using PRAS40^T246A^ plasmid DNA (Figure 4E).

In the absence of nutrients, PRAS40 binds to Raptor and inhibits mTOR kinase activity by blocking TOR signaling (TOS)-mediated substrate access to Raptor [25, 40]. Since IL-24^wt^ relieved PRAS40 from AKT inhibition, indicated by reduced pPRAS40^T246^ expression, we examined whether activated PRAS40 further interacted with Raptor to inhibit mTORC1. We observed less interaction between PRAS40 and Raptor in IL-24^wt^-expressing H1299 cells than in controls (Figure 5A). To determine whether our observation was common among the agents that disrupt mTOR, we tested rapamycin on H1299-*IL24^wt^* cells and observed reduced interaction between PRAS40 and Raptor in rapamycin-treated cells, compared with controls (Figure 5B). While we might speculate that IL-24^wt^ likely disrupts the mTOR complex, additional studies investigating the molecular mechanism are warranted.

Finally, we investigated the expression of proteins that are molecular targets of and regulated by AKT. Only IL-24^wt^ reduced the phosphorylation of GSK-3 and increased β-catenin phosphorylation (Figure 6). GSK-3 and β-catenin regulate cellular processes and are often deregulated in cancer [41]. The ability of IL-24^wt^ to modulate GSK-3 and β-catenin concurs with our previous report demonstrating that Ad-mediated IL-24 expression in breast cancer cells reduced the expression of these proteins [39]. The expression of cyclin D1, a cell-cycle regulator and a target gene of β-catenin, is increased in lung cancer and correlated with increased risk of tumor progression and metastasis [42, 43]. In the present study, only IL-24^wt^ reduced cyclin D1 expression (Figure 6). The ability of IL-24^wt^ to markedly inhibit β-catenin and cyclin D1 expression and activate GSK-3, determined by its phosphorylation, makes IL-24-based cancer therapy attractive.

Recent studies have shown that cyclin D1 interacts with and regulates FLNa expression in migrating breast cancer cells [44]. FLNa is a widely expressed molecular scaffold protein that organizes filamentous actin into networks and stress fibers [45], is involved in cell motility and invasion [46], and is frequently overexpressed in cancers, including lung cancer [46]. SiRNA-mediated knock-down of cyclin D1 reduced phosphorylation of FLNa at Ser2152 and Ser1459, resulting in reduced cell migration and invasion [44].

FLNa is also regulated by PAK1 [47]. PAKs play an important role in cellular processes, including cytoskeletal rearrangements and growth and apoptotic signal transduction through mitogen-activated protein kinases [47]. PAK1 phosphorylation at Threonine 423 by PDK1 is associated with an increase in phosphorylation of PAK1 substrates, including FLNa at Serine 2152 [48]. Physical interaction of FLNa with PAK1 has been shown to enhance PAK1 kinase activity, which subsequently phosphorylates FLNa at Serine 2152 [47, 48]. This bidirectional PAK1/FLNa interaction has been demonstrated to influence actin cytoskeletal structures and enhance tumor cell migration. Finally, PAK1 has been reported to be regulated by AKT and regulate chemotaxis [48]. Our study indicated that only IL-24^wt^ reduced pFLNa^S2152^and pPAK1^T423^ expression, coinciding with reduced AKT and cyclin D1 (Figure 6). We did not, however, analyze for cyclin B1, which has also been shown to regulate FLNa [49]. To our knowledge, this is the first report demonstrating that IL-24 protein phosphorylation is critical for modulating the AKT/mTOR signaling axis and exerting its anti-tumor activities in lung cancer. Whether IL-24-mediated inhibition occurs via the AKT/mTOR pathway in human cancers of different origins is unknown and should be investigated. Additionally, all of the experiments in this study were performed in a receptor-negative H1299 lung cancer cell line. Thus, the role of phosphorylation in IL-24 cytokine function in immune cells remains unexplored. Finally, the contribution of protein phosphorylation on IL-24 protein stability is not known and is currently being investigated in the laboratory.

In conclusion, we demonstrated that IL-24 phosphorylation is essential for its anti-cancer properties. IL-24 is the only IL-10 cytokine family member whose functions are regulated by PTMs, analogous to classical tumor suppressors. Our results provide a platform for identifying the phosphorylation site(s) that are critical for IL-24 to function as an anti-cancer drug.

## MATERIALS AND METHODS

### Cell culture

We cultured human non-small cell lung cancer cells (H1299) in RPMI 1640 medium (GIBCO) as previously described [4, 6]. The cell line was authenticated at the Genetic Resource Core Resource Facility, Johns Hopkins University, Baltimore, MD. In all experiments, untreated cells served as controls.

### IL-24 plasmid vectors and cell lines

*MDA-7/IL-24* (GenBank accession NM_006850, variant 1) cDNA is 621 bp in size with 206 amino acids. Potential phosphorylation sites of MDA-7/IL-24 protein predicted by NIH NCBI ORF finder are S88, S101, T111, T133 and S161 where S stands for Serine; T stands for Threonine. The software predicted that phosphorylation in MDA-7/IL-24 are as following: amino acid 88 SAR, amino acid 133 TLK, amino acid 161 SIR are casein kinase II consensus sites; amino acid 101 SDAE, amino acid 111 TLLE, amino acid 161 SIRD are protein kinase C (PKC) consensus sites [50].

Human *IL*-*24^wt^* cDNA cloned in pLJ143 plasmid backbone was used as a template to create the *IL-24* phosphorylation mutant (*IL-24^mt^*). Briefly, *IL*-*24^wt^* cDNA was retrieved by BamHI and XhoI restriction enzyme digestion and ligated back into to pBlueScript SK+ (Agilent). The sequence was verified by polymerase chain reaction (PCR) using primers *MDA-7*.F.BamHI (forward primer) and MDA7.R.XhoI (reverse primer; Table 1) and confirmed by DNA sequencing from T7 and M13R.

**Table 1:** PCR Primers used in “Overlay PCR” for generating *IL-24^mt^*

Prime Amino acid/Restriction E enzyme	Primer sequences
IL-24. F.BamHI	5′GCGGATCCGAGGAAGGCCAGGAGGAACAC 3′
IL-24. R.XhoI	5′ GCCTCGAGTGAAATGACACAGGGAACAAACCAGT 3′
IL-24- S88A.F.EagI	5′ GCTCAGGATAACATCACGGCCGCCCGGCTGCTGCAGCAG 3′
IL-24- S88A.R.EagI	5′ CTGCTGCAGCAGCCGGGCGGCCGTGATGTTATCCTGAGC 3′
IL-24- S101D.F.Hinc2	5′ GAGGTTCTGCAGAACGTCGACGATGCTGAGAGCTGTTAC 3′
IL-24- S101D.R.Hinc2	5′ GTAACAGCTCTCAGCATCGTCGACGTTCTGCAGAACCTC 3′
IL-24- T111Q.F.PvuII	5′ GAGCTGTTACCTTGTCCACCAGCTGCTGGAGTTCTACTTG 3′
IL-24- T111Q.R.PvuII	5′ CAAGTAGAACTCCAGCAGCTGGTGGACAAGGTAACAGCTC 3′
IL-24- T133Q.F.Pvu2	5′ GAACAGTTGAAGTCAGGCAGCTGAAGTCATTCTCTACTC 3′
IL-24- T133Q.R.Pvu2	5′ GAGTAGAGAATGACTTCAGCTGCCTGACTTCAACTGTTC 3′
IL-24- S161D.F.EcoRV	5′ CAAGAAAATGAGATGTTTGATATCAGAGACAGTGCACACAGG 3′
IL-24- S161D.R.EcoRV	5′ CCTGTGTGCACTGTCTCTGATATCAAACATCTCATTTTCTTG 3′

“F” and *“R”* preceding restriction enzyme denote forward and reverse primer respectively.

On the basis of wild-type *IL*-*24^wt^* cDNA sequence, *IL-24* phosphorylation mutant (*IL-24^mt^*) plasmid was generated by replacing the three serine (S) and two threonine (T) sites using the forward and reverse PCR primers (Table 1) and the “Overlay PCR” method. The primers designed (Table 1) for creating the *IL-24^mt^* was such that the amino acid replacing the native amino acid in MDA-7/IL24 protein had equivalent size, structural similarity, charge and polarity. In the “Overlay PCR” method, the forward primer was designed such that the intended one or two or three nucleotides which are to be replaced were flanked on each side with approximately 20 oligonucleotide bases. The forward primer was used in conjunction with a complementing reverse primer. The forward primer for a mutant is paired with a next 3′ reverse primer for a high fidelity PCR reaction; the reverse primer is paired with previous 5′ forward primer in a high fidelity PCR reaction. Then the two segments of PCR products, which share approximately 40 bases together (so-called overlay), are used as templates for overlaying both segments together using previous 5′ forward primer and next 3′ reverse primer. The resulting PCR product with all five phosphorylation site replaced was subsequently TA cloned into TOPO plasmid vector (Invitrogen) and sequenced at the DNA sequencing core facility (MD Anderson Cancer Center, Houston, TX).

The *IL-24^wt^* and *IL-24^mt^* cDNA from the TOPO plasmid vector was sub-cloned into pcDNA3.1+ plasmid expression vector (Invitrogen). The resultant plasmids were verified by BamHI-XhoI restriction enzyme digestion, PCR, and by the inserted signature restriction enzyme digestion. All PCR reactions performed in the study followed the High Fidelity PCR system protocol (Roche). Restriction enzymes used in the study were purchased from New England Biolabs. The pcDNA3.1+ plasmid vector carrying *IL-24^wt^* and *IL-24^mt^* cDNA sequence were either used in transient transfection studies or used in creating doxycycline (DOX)-inducible plasmid vector as described below.

### Tet-ON inducible IL-24 expression in H1299 lung cancer cell line

The Tet-ON inducible system was purchased from Clontech. The *IL-24^wt^* and *IL-24^mt^* cDNA sequence cloned in pcDNA3.1+ plasmid vector was retrieved by BamHI and PmeI restriction enzyme digestion and sub-cloned into BamHI and EcoRV enzyme digested pTRE-Tight plasmid (Clontech). The resulting pTre-*IL24^wt^* and pTre-*IL24^mt^* plasmids with appropriate inserts were verified by PCR, restriction enzyme digestions and signature enzyme digestions. The plasmids were subsequently used for creating Tet-ON inducible stable cell lines per manufacturer's guidelines. Briefly, human H1299 lung cancer cell line was transfected with pTet-ON-adv plasmid (Clontech) using Fugene (Roche) and selected in Neomycin (800 μg/ml; Sigma) for fourteen days. Transfection studies were performed using 10% tetracycline free fetal bovine serum (Atlanta Biologicals). The surviving fraction of cells was replated and transfected with pTreT-*IL-24^wt^* or pTreT-*IL-24^mt^* plasmid followed by hygromycin (400 μg/ml; Sigma) for two weeks. The surviving fraction of cells were then expanded and screened for IL-24^wt^ and IL-24^mt^ expression by Western blotting after DOX (1 μg/ml) treatment for 24 h. The IL-24^wt^ and IL-24^mt^ expressing cells were then seeded in ninety-six-well plates and subjected to single-cell cloning. Each clonal population of cells was subsequently screened for IL-24 protein expression by Western blotting. The clone that demonstrated the highest IL-24^wt^ and IL-24^mt^ protein expression was expanded and labeled as H1299-*IL24^wt^* and H1299-*IL24^mt^* respectively and used in all of the studies described herein.

### Cell viability assay

H1299-*IL24^wt^* and H1299-*IL24^mt^* cells (1 − 2 × 10^5^ cells/well) seeded in six-well plates were treated with DOX (1 μg/ml). The number of viable cells at 72 h after DOX treatment was determined by the trypan-blue exclusion assay method as described previously [6, 17].

### Colony-forming assay

H1299-*IL24^wt^* and H1299-*IL24^mt^* cells (2 × 10^4^) were mixed with 0.7% low-melting agarose and seeded in six-well tissue culture plates previously coated with 0.7% agarose. The plates were incubated at 37°C for 24 h. Then, the cells were treated with DOX (1 μg/ml). Incubation continued for fourteen days at 37°C, with culture medium replenished twice a week. The number of surviving colonies was determined by staining with 0.005% crystal violet. Colonies were photographed and counted.

### Cell cycle analysis

H1299-*IL24^wt^* and H1299-*IL24^mt^* cells (1 × 10^5^) seeded in six-well plates were treated with DOX (1 μg/ml). Untreated cells served as controls. Cells were collected at 48 h after treatment and subject to flow-cytometric analysis as previously described [6, 17].

### Western blotting assay

H1299-*IL24^wt^* and H1299-*IL24^mt^* cells (1 × 10^5^/well) seeded in six-well plates were trypsinized and collected at 24 h and/or at 48 h after DOX (1 μg/ml). Cell lysates were prepared and protein samples subjected to western blotting [6, 11, 17]. Primary antibodies against IL-24 (Introgen Therapeutics), pAKT^S473^, pAKT^T308^, AKT, pAKT1^S473^, AKT1, pAKT2^S474^, AKT2, pPRAS40^T246^, PRAS40, pmTOR^S2448^, mTOR, pGSK-3^S21/9^, GSK-3, pβ-catenin S33/37/T41, β-catenin, pFLNa^S2152^, FLNa, pPAK1^T423^, PAK1, pJNK^Thr183/Tyr185^, JNK, cyclin D1, caspase-9, and PARP (Cell Signaling) were purchased and used. Beta-actin (Sigma) was used as an internal loading control. Proteins were detected using the appropriate secondary antibodies, as previously described [6, 11, 17, 39]. Protein expression levels were quantified using Image Quant (Syngene) software.

### Immunoprecipitation assay

Cells (5 × 10^6^) actively growing in 150-mm tissue culture plates were treated with DOX (1 μg/ml). Forty-eight hours after treatment, the cells were washed and trypsinized, and total cell lysates prepared. About 500 μg of total protein was mixed with 100 μl of appropriate antibody (phospho-Serine, phospho-Threonine, PRAS40; Cell Signaling) and incubated at 4°C overnight with constant mixing. Immune complexes were captured by incubation with 75–100 μl protein A/G-coated agarose beads for 1 h at 4°C. The immunoprecipitated proteins were recovered by boiling the agarose beads in 2X SDS sample buffer and western blotting. The membrane was subsequently probed using the appropriate primary antibody (IL-24, Raptor), and the protein bands detected as previously described [6, 17].

### Immunofluorescence assay

H1299-*IL24^wt^* and H1299-*IL24^mt^* cells (1 × 10^3^) seeded in two-well glass chamber slides were treated with DOX (1 μg/ml). Twenty-four hours after treatment, the cells were washed with 1.0 mL warm 1X phosphate-buffered saline (PBS) at 37°C and subsequently fixed in 4% paraformaldehyde in PBS for 10 min at room temperature (RT). After fixation, the cells were washed with PBS, incubated with 100% methanol for 10 min, and permeabilized with 0.5% TritonX-100 for 10 min. The cells were washed with PBS, followed by incubation in blocking buffer (3% BSA + 1% normal serum) in PBS for 1 h at RT. Then, we added a mixture of anti-human IL-24 antibody raised in goat (5.0 μl; Introgen Therapeutics) and mouse anti-PDI antibody (0.5 μl; Molecular Probes) in blocking buffer and incubated for 2 h at RT. The cells were subsequently washed with 0.5 ml of 1X blocking buffer, followed by the addition of a mixture of Alexa Fluor^®^ 488 (anti-mouse green) and Alexa Fluor^®^ 594 (anti-goat red) diluted in 1 ml of blocking buffer. The cells were incubated in the dark for 1 h at RT, then counter stained with DAPI (Sigma) for 1 min. The stained cells were washed with 1X PBS, mounted with Dako cytomation fluorescent mounting media (Dako), and then viewed using a laser confocal microscope equipped with appropriate fluorescein filters.

### Enzyme-linked immunosorbent assay (ELISA)

H1299-*IL24^wt^* and H1299-*IL24^mt^* cells (1 × 10^4^) seeded in six-well plates were incubated overnight at 37°C, 5% CO_2_. The next day, the culture medium was replaced with fresh RPMI-1640 medium containing 2% FBS. DOX (1 μg/ml) was added. Culture supernatants were collected at different times after treatment, centrifuged, and stored at −80°C. We detected IL-24 protein in the supernatant by sandwich ELISA (R&D Systems). Briefly, ninety-six well flat-bottomed plates were coated with 100 μl of anti-IL-24 capture antibody (2.0 μg/mL; R&D Systems) overnight at +4°C. The plates were washed with sterile PBS followed by incubation in 100 μl of blocking buffer containing 10% bovine serum albumin (BSA in PBS) for 1 h at RT. On completion of incubation, the blocking buffer was aspirated and 100 μl of culture supernatant collected from H1299-*IL24^wt^* and H1299-*IL24^mt^* cells with and without DOX treatment was diluted in PBS (1:2) and added in duplicate wells and incubated for 2 h at RT. The culture medium from the wells was subsequently aspirated and washed with sterile PBS. To the wells 100 μl of horse-radish peroxide (HRP)-conjugated anti-IL-24 detection antibody (R&D Systems) was added. After 1 h incubation, the wells were washed with sterile PBS followed by addition of streptavidin-horse radish peroxidase (100 μl/well) and incubation in the dark for 20 min at RT. The reaction was stopped by adding 50 μl of stop solution (R&D Systems). The plates were then read using a plate reader equipped with 450 nm excitation and 540 nm emission filter.

### *In vitro* scratch assay

The *in vitro* scratch assay was performed using the Cytoselect^TM^ 24-well wound healing assay (Cell Biolabs). Briefly, H1299-*IL24^wt^* and H1299-*IL24^mt^* cells (2.5 × 10^4^) were added to 24-well plates with inserts in place. After 24 h incubation, we removed the inserts to generate a 0.9 mm wound field, washed it with media, and incubated at 37°C. The cells were then treated with DOX (1 μg/ml). We photographed the scratch area 24 h later using a Nikon TiU microscope (Nikon). The distances between the two cell edges were measured per the manufacturer's instructions.

### Cell migration assay

H1299-*IL24^wt^* and H1299-*IL24^mt^* cells (5 × 10^4^) suspended in 1 ml RPMI-1460 medium were seeded in the upper chambers (8 μm; BD Biosciences) and placed in a six-well plate filled with serum-free RPMI-1640 medium (lower chamber). After 24 h, the culture medium was replaced with fresh medium containing 20% tetracycline-free FBS (Atlanta Biologicals). The upper chamber was filled with 2% tetracycline-free FBS-containing medium, with or without DOX (1 μg/ml; Sigma). Following incubation for 24 h and 48 h, the inserts were removed and processed as previously described [20, 51, 52]. Four random fields were selected and we counted the migrated cells per field under an inverted bright-field microscope at 10X magnification. The results were expressed as the average number of cells of four fields.

### Cell invasion assay

The cell invasion assay was performed using six-well Matrigel invasion chambers with 8-μm pore inserts (BD Biosciences). H1299-*IL24^wt^* and H1299-*IL24^mt^* (5 × 10^4^) cells were seeded into the upper chambers. The lower chambers were filled with serum-free medium. After 24 h, the medium was replaced with 20% FBS-containing culture medium, and the upper chambers were filled with 2% FBS-containing culture medium, with or without DOX (1 μg/ml). After an additional 24 h and 48 h incubation, the filters were removed and processed as previously described [20, 51, 52]. Tumor cell invasiveness was determined by counting cells in four microscopic fields per well. The extent of invasion was expressed as an average number of cells per field.

### RNA interference studies

H1299-*IL24* (wt and mt) cells (1 × 10^5^ cells/well) were seeded in serum-free medium in six-well plates and transfected with 100 nM AKT siRNA (Santa Cruz) using transfection reagent (Dharmacon), per the manufacturer's instructions. Six hours later, the medium was replaced with RPMI-1460 containing 2% tetracycline-free serum, with or without 1 μg/ml DOX. Untransfected cells served as a control. After 48 h of incubation, the cells were harvested and total cell lysates prepared for western blot analysis.

For the cell migration assay, H1299-*IL24* (wt and mt) cells (5 × 10^4^) were seeded in the upper chamber of the inserts and transfected with AKT siRNA (100 nM), with or without 1 μg/ml DOX. The lower chamber was filled with 20% tetracycline-free FBS-containing medium. We counted the number of migrated cells 48 h after treatment.

### AKT and PRAS40 overexpression studies

H1299-*IL24^wt^* cells (1 × 10^5^ cells/well) were seeded in six-well plates and transfected with 0.5 μg myr-*AKT* or pPRAS40^T246^ DNA (Addgene) using Lipofectamine 2000 (Invitrogen), per the manufacturer's instructions. Six hours after transfection in serum-free medium, the medium was replaced with RPMI-1460 containing 2% tetracycline-free serum, with or without 1 μg/ml DOX. Untransfected cells served as controls. After 48 h of incubation, the cells were harvested and total cells lysate subjected to western blotting.

For the cell migration assay, H1299-*IL24^wt^* cells (5 × 10^4^) were seeded in the upper chamber of the inserts and were transfected with myr-*AKT* plasmid (0.5 μg), with or without 1 μg/ml DOX. The rest of the procedure followed the description under “cell migration assay”.

### Statistical analysis

All experiments were performed at least twice. Unless otherwise stated, all data are shown as mean ± *SD*. Data were analyzed using two-tailed student's *t* test or one-way analysis of variance (ANOVA) using SPSS 12.0 (SPSS, Inc., Chicago, IL). *P* < 0.05 was considered statistically significant.

## SUPPLEMENTARY FIGURE


