# Supplementary material for: Phosphorylation of interleukin (IL)-24 is required for mediating its anti-cancer activity
Source: Oncotarget. 2015 May 18;6(18):16271–86. doi: 10.18632/oncotarget.3977 (PMC4599269; doi:10.18632/oncotarget.3977)
Supplement: Supplementary file 1 [file oncotarget-06-16271-s001.pdf]

SUPPLEMENTARY FIGURE

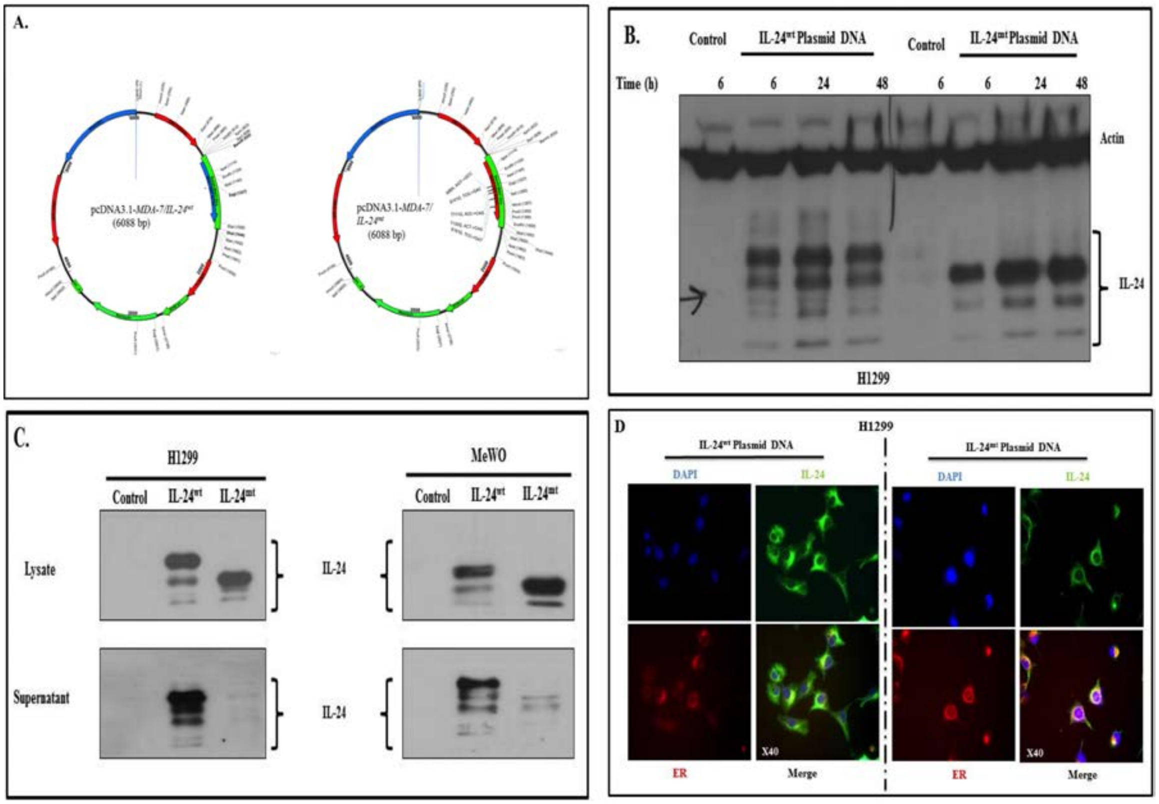

Supplementary Figure S1: Difference in IL-24^{wt} and IL-24^{mt} protein expression is not unique to doxycycline-inducible stable cell line. A. Map of pcDNA3.1+ plasmid expression vector carrying the *IL-24^{wt}* and *IL-24^{mt}* cDNA sequence under the control of constitutively active cytomegalovirus (CMV) promoter. **B.** Naïve H1299 cells were transiently transfected with a pcDNA3.1 + -*IL-24^{wt}* or -*IL-24^{mt}* plasmid DNA and subjected to Western blotting as described in the "Methods" section. IL-24^{wt} and IL-24^{mt} protein banding pattern observed was similar to that observed in DOX-treated H1299-*IL-24^{wt}* and H1299-*IL-24^{mt}* cell lines. Beta actin was detected for protein loading control. Cells that were not transfected served as control. **C.** IL-24^{wt} and IL-24^{mt} protein expression in human melanoma (MeWo) cell line that was transiently transfected with the plasmid DNA as described above in "B" showed protein banding patterns similar to that observed in H1299 cells. Strong IL-24^{wt} protein expression was detectable in both the cell lysate and supernatant while IL-24^{mt} protein was detectable only the cell lysate with a very faint protein band detectable in the supernatant. **D.** Naïve H1299 cells transfected with pcDNA3.1+-*IL-24^{wt}* plasmid showed diffuse cytoplasmic staining of IL-24 with some staining in the ER. In pcDNA3.1 + -*IL-24^{mt}* plasmid transfected H1299 cells, the IL-24 protein staining was primarily observed in the ER with some staining observed in the cytoplasm.
